# Hen raising helps chicks establish gut microbiota in their early life and improve microbiota stability after H9N2 challenge

**DOI:** 10.1186/s40168-021-01200-z

**Published:** 2022-01-24

**Authors:** Xiaobing Li, Ran Bi, Kangpeng Xiao, Ayan Roy, Zhipeng Zhang, Xiaoyuan Chen, Jinyu Peng, Ruichen Wang, Rou Yang, Xuejuan Shen, David M. Irwin, Yongyi Shen

**Affiliations:** 1 Center for Emerging and Zoonotic Diseases, College of Veterinary Medicine, South China Agricultural University, Guangzhou, 510642 China; 2 Guangdong Laboratory for Lingnan Modern Agriculture, Guangzhou, 510642 China; 3 Department of Biotechnology, Lovely Professional University, Bengaluru, India; 4 Zhaoqing Branch Center of Guangdong Laboratory for Lingnan Modern Agricultural Science and Technology, Zhaoqing, 526238 China; 5 Department of Laboratory Medicine and Pathobiology, University of Toronto, Toronto, M5S1A8 Canada; 6 Banting and Best Diabetes Centre, University of Toronto, Toronto, M5S1A8 Canada; 7 Guangdong Provincial Key Laboratory of Zoonosis Prevention and Control, Guangzhou, China

**Keywords:** Gut microbiota, Maternal microbial transmission, H9N2 avian influenza virus, Disease resistance

## Abstract

**Background:**

Early gut microbial colonization is important for postnatal growth and immune development of the chicken. However, at present, commercial chickens are hatched and raised without adult hens, thus are cut off from the microbiota transfer between hens and chicks. In this study, we compared the gut microbiota composition between hen-reared and separately reared chicks, and its impact on the resistance to H9N2 avian influenza virus, with the motive of investigating the impact of this cutoff in microbiota transfer.

**Results:**

We used the 16SrRNA sequencing method to assess the composition of the gut microbiota in chicks represented by three hen-reared groups and one separately reared group. We found that the diversity of gut microbes in the chicks from the three hen-reared groups was more abundant than in the separately reared group, both at the phylum and genus levels. Our findings highlight the importance of early parental care in influencing the establishment of gut microbiota in the early life of chicks. SourceTracker analysis showed that the feather and cloaca microbiota of hens are the main sources of gut microbiota of chicks. After H9N2 exposure, the viral infection lasted longer in the separately reared chicks, with the viral titers in their oropharyngeal swabs being higher compared to the hen-reared chicks at day 5 post-infection. Interestingly, our results revealed that the gut microbiota of the hen-reared chicks was more stable after H9N2 infection in comparison to that of the separately reared chicks.

**Conclusions:**

Microbiota transfer between the hens and their chicks promotes the establishment of a balanced and diverse microbiota in the early life of the chicks and improves microbiota stability after H9N2 challenge. These findings advance our understanding of the protective role of gut microbiota in the early life of chicks and should be instrumental in improving chick rearing in the commercial poultry industry.

Video Abstract

**Supplementary Information:**

The online version contains supplementary material available at 10.1186/s40168-021-01200-z.

## Background

The complex microbiome on and within a host, critically linked with health and well-being, is referred to as the “second genome” of the host [1]. This “second genome” is not innate, but is derived from microbial dispersal and affects the growth [2, 3], immunity [4], behavior and cognitive abilities of the host [5, 6].

Maternal factors present in the prenatal and postnatal environments are important for gut microbial assembly in the offspring [7]. There is mounting evidence suggesting that the establishment of the gut microbiota in early life is dependent on mother-to-child transmission. For example, before the birth of a fetus, compounds produced by the mother’s microbiota can be transferred to the fetus to promote the generation of innate immune cells [8]. As the fetus passes through the birth canal, maternal vaginal microorganisms are ingested and vertically transferred [9]. After birth, a variety of microorganisms from the mother and the environment further colonize the gut of the newborn [9]. These processes are crucial for the recruitment and establishment of the neonatal microbiota and promote the development of the immune system in the gut, and other parts of the body, for defense against pathogens [10]. Babies born by cesarean delivery and fed formula have a delayed development of their gut microbiota due to insufficient contact with their mothers, which slows the maturation of the metabolic and immune systems [11–13].

In oviparous animals (such as birds), mechanisms associated with the establishment and assembly of early-life gut microbiota may be different from those observed in mammals [14]. Specifically, embryos develop in closed eggs and experience minimal contact with microbes from ovipositors, maternal feathers, and other components of the nest environment [15–17]. After hatching, parental care behaviors that include egg incubation, saliva exchange between parents and nestlings during feeding, and prolonged periods of physical contact between the parents and offspring, facilitate the transmission of gut microbiota and subsequent colonization in chicks [18, 19].

Recent studies have emphasized the imperative role of the gut microbiota in shaping immunity against viral diseases in chickens and ducks [20, 21]. However, the influence of the maternally mediated early-life assembly of the gut microbiota on the immune system in birds remains mostly unexplored. A study reported low gut microbial diversity in hand-reared passerine chicks and inferred that parental care is crucial in shaping the gut microbiota of the chicks [19]. However, the impact of microbiota transfer and establishment in the chicks, and its role in disease resistance demands to be investigated at deeper insights. As an important farm animal, chickens have been hatched and fed by hens throughout their evolutionary history. However, recent commercial chicken breeding is based on hatcheries and the chicks are raised in the absence of adult hens. The consequences of this switch in the feeding mode on the gut microbiome and associated immunity need to be addressed.

In this study, we examined and compared the gut microbiota composition and dispersal in hen-reared and separately reared chicks under laboratory conditions. Furthermore, we compared the resistance of these two groups of chicks against the H9N2 avian influenza virus (AIV) to evaluate the effect of the maternally mediated early-life gut microbiome assembly in immune development. Our research systematically studied the influence of maternal factors on the establishment of early-life gut microbiota and immune system development in chickens, which helps deepen our understanding of the healthy development of chickens in the poultry industry.

## Results

### Hen-reared chicks had higher gut microbiota diversity

**Fig. 1:**
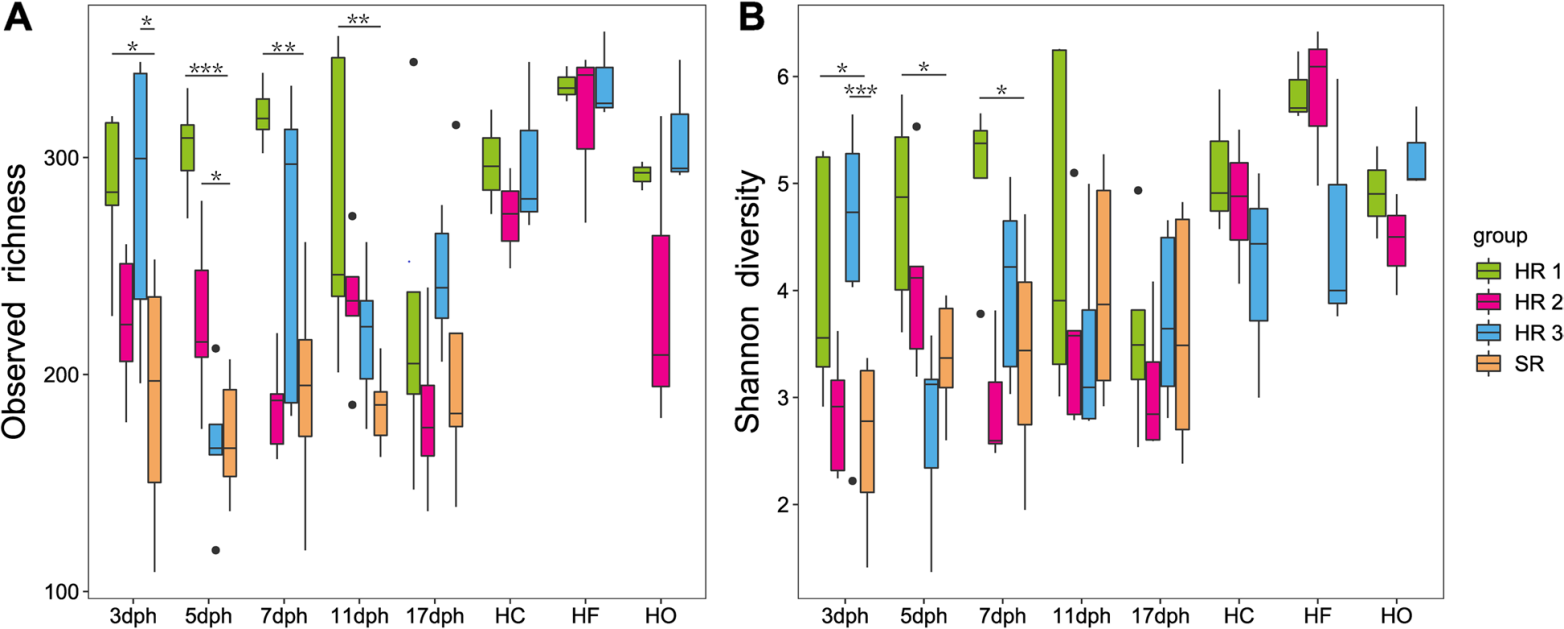
Boxplots showing the alpha diversity indices of gut microbiota from hen-reared (HR) chicks, separately reared (SR) chicks, and hens. **A** Observed richness and **B** Shannon diversity index. The horizontal bar in the boxes represents the median. The top and bottom of the boxes represent the 75th and 25th percentiles, respectively. The upper and lower whiskers extend to data not exceeding 1.5× the interquartile range from the upper edge and lower edge of the box, respectively. The asterisk represents significant difference in comparison to the separately reared group by the Dunnett test, *P < 0.05; **P <0.01;***P < 0.001. dph: days post-hatching; HC: hen cloacal swab; HF: hen feathers; HO: hen oropharyngeal swab

Three hen-reared (HR) groups and one separately reared (SR) group of chicks were designed for this study (Table S1**)**. To determine whether maternal symbiotic microbes influence the gut microbiota diversity of the chicks, alpha diversity of gut microbiota in the chicks was estimated by the Shannon and Observed OTUs index (Fig. 1A, B and Table S2). Our results revealed that from days 3 to 5 post-hatching (dph), generally, gut microbiota diversity of hen-reared groups was significantly higher than that of chicks in the separately reared group (Dunnett test, *P* < 0.05). Gut microbiota richness of the hen-reared groups was significantly higher than the separately reared group at 3 to 11 dph (Dunnett test, *P* < 0.05). Age in days had an effect on the diversity and richness in the gut microbiota of the chicks of the separately reared group, with diversity and richness showing initial increase between 5 to 7 dph, followed by a subsequent decrease. For the maternal bacterial community, on average, feathers of the hens had a higher richness and diversity than the oropharyngeal and cloacal swabs (Fig. 1).

### Hen-reared chicks had a richer initial gut microbiota composition

**Fig. 2:**
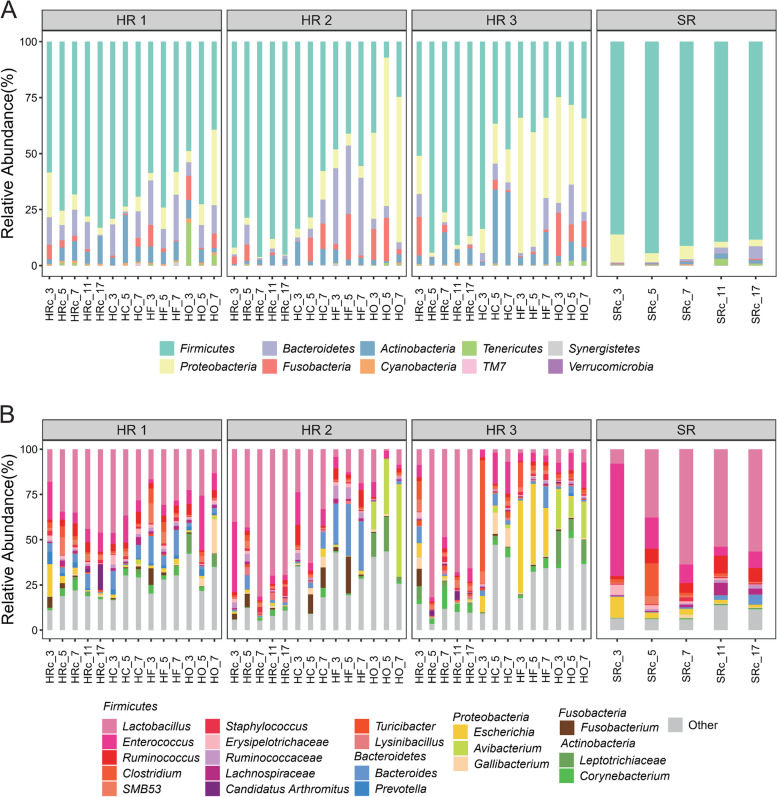
Composition of the gut microbiota among the different groups. **A** Phylum-level and **B** top 20 common genus-level gut microbial communities in the hen-reared (HR) chicks, separately reared (SR) chicks, and maternal hen microbiota. HC: hen cloacal swab; HF: hen feathers; HO: hen oropharyngeal; HRc: hen-reared chick; SRc: separately reared chick

To investigate the establishment and variation of gut microbiota during chick development, we statistically analyzed the composition and abundance of bacterial taxa in the microbiota of chicks and hens. The microbial compositions of feather, oropharyngeal, and cloacal swabs of the hens are quite different. For example, at the phylum level (Fig. 2A), oropharyngeal swab samples from most of the hens had higher levels of *Proteobacteria* (average of 38.04%). In contrast, *Firmicutes* (feather: 49.36%; cloacal swab: 67.80%), *Bacteroidetes* (feather: 18.45%; cloacal swab: 6.41%), and *Actinobacteria* (feather: 6.15%; cloacal swab: 13.07%) were more prevalent in the feather and cloacal swabs. At the genus level (Fig. 2B), oropharyngeal swab samples from hens displayed abundance in *Avibacterium* (16.48%) and *Leptotrichiaceae* (9.74%), while feather samples had higher proportions of *Bacteroides* (13.99%) and *Lactobacillus* (13.27%). The cloacal swab samplers were noted to be enriched with *Lactobacillus* (26.71%), *Enterococcus* (10.88%), and *Clostridium* (7.82%).

In the separately reared group, the composition of the gut microbiota of the chicks had high relative abundances of *Firmicutes* (86.16%, 95.5%, 91.27%) and *Proteobacteria* (12.45%, 4.08%, 5.91%) at 3–7 dph, with the relative abundances of other phyla being less than 1%. From 11 to 17 dph, a slight increase was noted in the abundances of *Bacteroidetes* (11 dph: 2.61%, 17 dph: 5.6%), *Actinobacteria* (11 dph: 2.26%, 17 dph: 1.62%) and *Tenericutes* (11 dph: 2.91%, 17 dph: 0.67%) in the separately reared group (Fig. 2A). At the genus level (Fig. 2B), *Enterococcus* (62.12%) was observed to be most abundant in the separately reared chick group at 3 dph, which decreased at 5 dph (17.26%), whereas *Lactobacillus* (37.80%), *Clostridium* (18.10%), and *Ruminococcus* (8.09%) increased. The gut microbiota was generally stable between 7 to 17 dph and was dominated by *Lactobacillus* (63.72–56.65%), followed by *Ruminococcus* (5.33–7.63%) and *Enterococcus* (10.31–8.98%).

Chicks in the hen-rearing group had more abundant gut microbiota, with high abundances of *Firmicutes* at 3 to 7 dph, including other phyla that were common in the hen gut communities — *Bacteroidetes* (3dph, HR 1–3: 12.22%, 1.02%, 10.23%; 5 dph, HR 1–3: 6.79%, 8.82%, 0.25%), *Proteobacteria* (3 dph, HR 1–3: 20.04%, 2.99%, 17.07%; 5 dph, HR 1–3: 6.47%, 3.10%, 1.73%), *Actinobacteria* (3 dph, HR 1–3: 2.08%, 0.49%, 3.75%; 5 dph, HR 1–3: 6.01%, 1.26%, 3.33%), and *Fusobacteria* (3 dph, HR 1–3: 6.52%, 3.27%, 17.23%; 5dph, HR 1–3: 3.25%, 7.37%, 0.09%) (Fig. 2A). At the genus level, chicks in the hen hen-reared groups were found to be generally enriched with *Lactobacillus* (HR 1–3: 18.06%, 40.15%, 8.72%), *Enterococcus* (HR 1–3: 20.86%, 38.90%, 6.82%), *Fusobacterium* (HR 1–3: 6.10%, 3.11%, 9.52%), and *Bacteroides* (HR 1–3: 4.69%, 0.92%, 8.81%) at 3 dph; and enriched with *Lactobacillus* (HR 1–3: 34.59%, 43.32%, 81.91%), *SMB53* (HR 1–3: 9.72%, 4.09%, 2.35%), and *Bacteroides* (HR 1–3: 3.24%, 7.88%, 0.23%) at 5 dph.

### Difference in the gut microbiota of chicks under different feeding modes

**Fig. 3:**
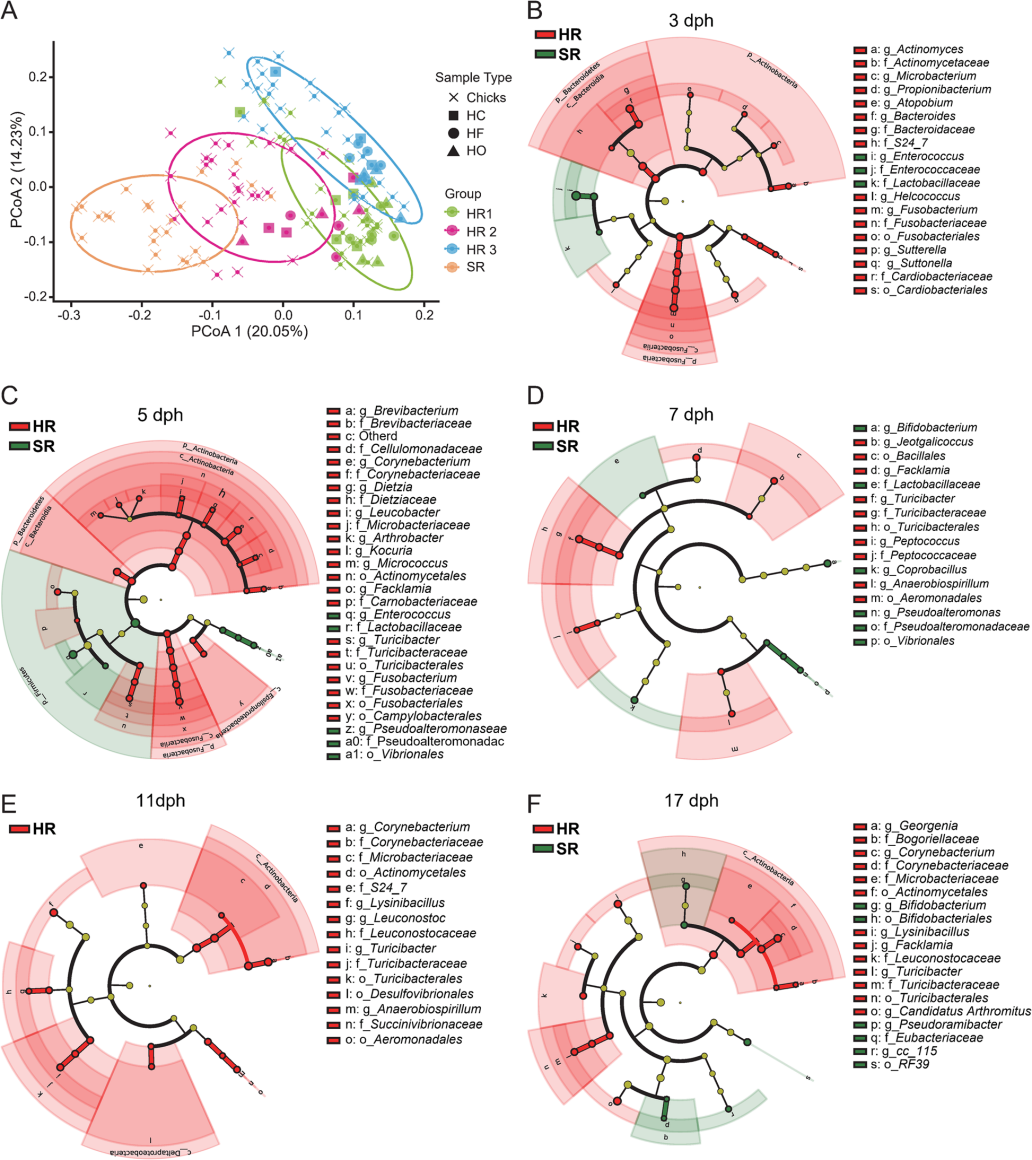
Differences in the gut microbiota of chicks raised under different rearing patterns. **A** Principal coordinates analysis (PCoA) of the bacterial communities from the four reared group chicks and their hens. HC: hen cloacal swab; HF: hen feathers; HO: hen oropharyngeal. **B**–**F** LEfSe analysis generated differences in the abundance of the bacterial taxa of hen-reared groups (red) and separately reared group (green) chicks at different times (*P* < 0.05, LDA > 2)

A principal coordinate analysis (PCoA), based on the Bray–Curtis distances, was used to assess the differences in bacterial community structure between the chick and the hen microbiome samples (Fig. 3A). The PCoA revealed that the samples of the hen-reared (HR) chicks were closer to their mothers. Our observations indicated that the gut microbiota of chicks in each group was influenced by the maternal bacterial communities. Moreover, the distance between the three hen-reared groups was closer (ANOSIM, *R* = 0.4748, *P* = 0.001).

Linear discriminant analysis effect size analysis (LEfSe) (*P* < 0.05, LDA > 2) identified representative gut bacterial taxa for the chicks in the hen-reared groups and the separately reared group. Consistent with the microbial composition results, the diversity of gut microbiota varied greatly in the early life of the chicks. At 3 to 5 dph (Fig. 3B, C), phyla *Actinobacteria*, *Bacteroidetes*, and *Fusobacteria* and their classified bacteria were the dominant genera in the hen-reared group, with only families *Enterococcaceae* and *Lactobacillaceae* being significantly enriched in the separately reared group. At 11 dph (Fig. 3E), no dominant microbiota genera were found in the separately reared group, whereas the gut microbiota of the hen-reared groups was enriched in the order *Actinomycetales*, genus *Lysinibacillus*, and families *Muribaculaceae* (*S24-7*), *Leuconostocaceae*, and *Turicibacteraceae*. At 17 dph (Fig. 3F), the family *Eubacteriaceae*, genus *Erysipelotrichaceae* CC-115, order *Bifidobacteriales*, and *Enterococcaceae* bacterium RF39 were dominant in the separately reared group, whereas the family *Corynebacteriaceae*, genus *Lysinibacillus*, and orders *Actinomycetales* and *Turicibacteriales* were prevalent in the chicks of the hen-reared groups.

### Maternal gut microbes are substantial contributors to the gut microbiota of chicks in early life

**Fig. 4:**
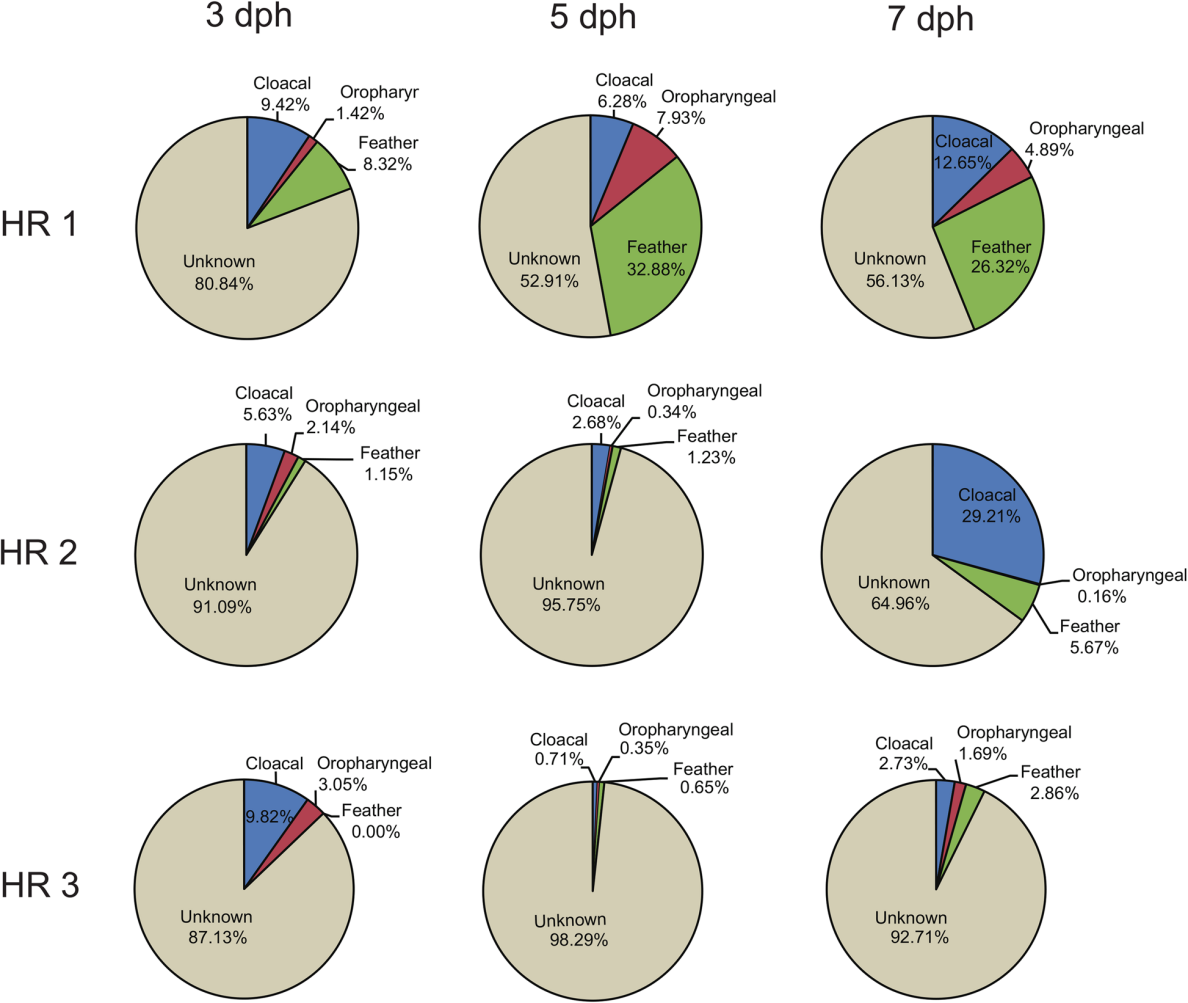
Community-level source-tracking models for the maternal sources of early gut community assembly. Pie charts of the mean proportions

It was evident from our studies that the gut microbiota of chicks and their maternal hen’s microbiota had a considerable OTU-level similarity. Accordingly, we used Bayesian community-level source tracking to investigate the contribution of maternal sources in the gut community assembly of the chicks. The SourceTracker analysis predicted that the maternal cloacal swab and feather communities were important sources for the gut communities of the chicks (Fig. 4). At 3 dph, the chick gut microbiota communities of the three hen-reared groups were predicted to have received 9.42%, 5.63%, and 9.82% of their bacteria from respective hen cloacal swab communities, with an additional 1.15–9.32% from their hen’s feather communities. At 5 dph, the maternal microbiota of hen-reared groups 1 and 2 contributed had lower contribution to the chick’s gut microbiota, although the maternal microbiota of hen-reared group 1 was still the main source of the gut microbiota of its chicks. At 7 dph, the gut microbiota of the three hen-reared groups 1, 2, and 3 were 12.65%, 29.21%, and 2.73% from the cloaca of their hens, and 26.32%, 5.67%, and 2.86% from the hen’s feather, respectively. In contrast, the maternal oropharyngeal microbiota was not a major source of the gut microbiota for the chicks at any stage.

### Hen-reared chicks showed a more stable bacterial community composition after H9N2 infection

**Fig. 5:**
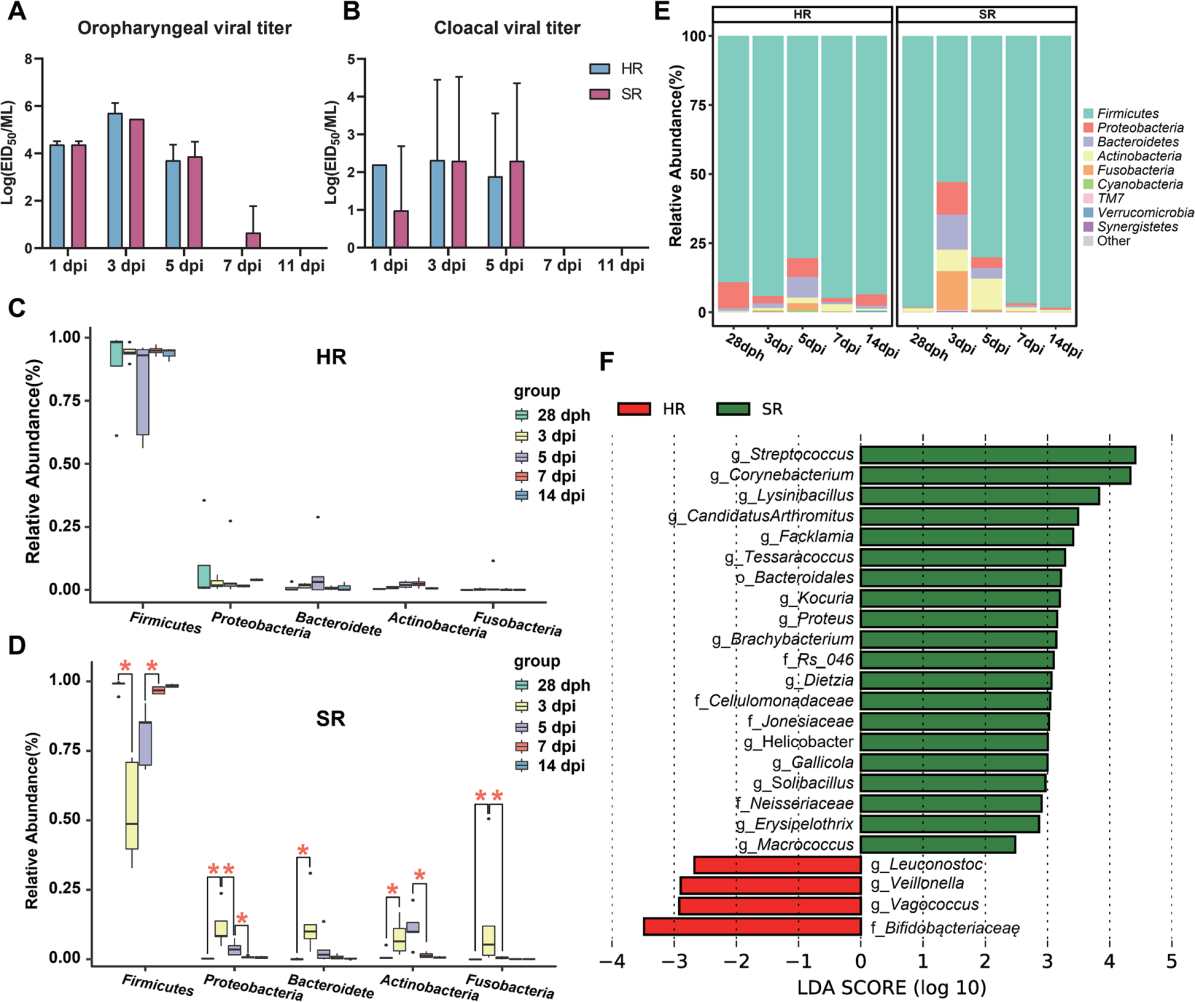
Viral titers and gut microbiota of chicks infected with H9N2 virus in the hen-reared (HR) and separately reared (SR) groups of chicks. **A**, **B** Viral titers in oropharyngeal and cloacal swabs of post-H9N2-infected chickens. Chicks (28 dph) were intranasally inoculated with 10^−6^EID_50_/0.2ml of LPAIH9N2. EID_50_ was calculated by the Reed and Muench method. **C**, **D** Comparison of the relative abundances of the major bacterial phyla representing the gut microbiota of HR and SR groups after H9N2 infection; *P < 0.05; the horizontal bar in the boxes represents the median. The top and bottom of the boxes represent the 75th and 25th percentiles, respectively. The upper and lower whiskers extend to data not exceeding 1.5× the interquartile range from the upper edge and lower edge of the box, respectively. **E** Phylum-level composition of the gut microbiota in the post-H9N2-infected chickens. **F** LEfSe analysis of the 16SrRNA sequences of the post H9N2 infected chickens (*P* < 0.01, LDA > 2)

We predicted the functional profiles of the gut microbiota from the chicks using PICRUSt [22] and found that the pathways associated with membrane transport, amino acid metabolism, carbohydrate metabolism, and replication and repair were enriched in the hen-reared chicks. Most of these functional pathways can be linked to the basic life activities of the chicken. For the separately reared chicks, there was a greater enrichment of pathways associated with disease, such as infectious diseases (Figure S1). These results offered the scope to speculate that chicks with different rearing patterns might have differing resistance to infectious diseases. To assess the impact of hen rearing on disease resistance in chicks, we performed an infection experiment with the H9N2 avian influenza A virus (AIV). We selected the HR2 group of the hen-reared group of chicks (here in referred to as hen-reared group) and the separately reared group of chicks to infect with the H9N2 virus. At days 1, 3, 5, 7, and 11 post-infection (dpi), viral titers from oropharyngeal and cloacal swabs were determined (Fig. 5A, B). In both groups, the peak of viral shedding detected by oropharyngeal swabs was at 3 dpi with a decline at 5 dpi. The separately reared group of chicks still had detectable virus in one oropharyngeal swab at 7 dpi (1.95 lgEID50/mL, average of 0.65 lgEID50/mL). The viral titers assessed by cloacal swabs in the separately reared group (3.45 ± 0.71 lgEID50/mL) of chicks were slightly higher than in the hen-reared group (2.82 ± 0.53 lgEID50/mL) at 5 dpi, though this difference was not statistically significant.

The bacterial community compositions of the cloacal swabs collected on 3, 5, 7, and 14 dpi were assessed by sequencing amplicons of the 16S rRNA gene. Before H9N2 challenge, our results showed that there were significant differences in the composition of the gut microbiomes between the hen-reared group (HR2 group) and the separately reared group (SR group) before 7dph, but there is no significant difference after 7dph (Table S3). For example, the family *Fusobacteriaceae* of the hen-reared group was significantly more abundant than in the separately reared group at 3 to 5 dph (Mann-Whitney *U* test, *P* < 0.05) (Table S3). There was no significant difference in the diversity (Dunnett test, *P* > 0.05) and composition (Mann-Whitney *U* test, *P* > 0.05) of gut microbiota between the separately reared group and hen-reared group at the day of the challenge (28 dph) (Table S2, Table S3). After H9N2 challenge, microbial classifications revealed that the composition of the chick microbiota changed more dramatically in the separately reared group after H9N2 challenge (Fig. 5C, D, and E). Within these chicks, *Firmicutes* were significantly reduced and *Proteobacteria*, *Fusobacteria*, *Bacteroidetes*, and *Actinobacteria* were significantly increased (Mann-Whitney *U* test, 28 dph vs 3 dpi, *P* < 0.05) at 3 dpi, whereas *Fusobacteria* and *Proteobacteria* were significantly reduced in the separately reared group at 5 dpi (Mann-Whitney *U* test, 3dpi vs 5 dpi, *P* < 0.05) (Fig. 5D, Table S4). At 7 dpi, *Proteobacteria* and *Actinobacteria* were significantly reduced and *Firmicutes* were significantly increased in the separately reared group (Mann-Whitney *U* test, 5dpi vs 7 dpi, *P* < 0.05) (Fig. 5D, Table S4). In contrast, the composition of the chick microbiota in the hen-reared group did not change significantly at the phylum level from 3 to 14 dpi (Fig. 5D, Table S4). Overall, the composition of chick microbiota in the hen-reared groups was observed to be more stable after H9N2 challenge (Fig. 5C, Table S4). Supervised comparison of samples using the LEfSe algorithm and logarithmic LDA (*P* < 0.01, LDA > 2) showed that in the hen-reared group, the cloacal swab microbiota was characterized by genera *Vagococcus*, *Veillonella*, and *Leuconostoc* and the family *Bifidobacteriaceae*, while the separately reared group were differentially enriched with the pathogenic bacterium genera *Streptococcus* and *Corynebacterium*, as well as *Lysinibacillus*, *Facklamia*, and *Tessaracoccus* (Fig. 5F).

## Discussion

The immune system of animals rapidly develops early in life and is affected by the gut microbiome [23]. Gut microbiota in early life imprints the host immune phenotype for a long time and affects the ability to resist diseases in later phases of life [24]. Current poultry industry chick-rearing methods disrupt microbiota transmission between hens and their chicks. We explored the influence of maternal microbial sources on the early development of the chick gut microbiota in this study. Hen-reared chicks had a diverse gut microbiota, while the gut microbiome of the separately reared chicks had a much lower diversity and richness in the first few days after hatching. Our results are consistent with studies in mammals that suggest the high microbial diversity of gut communities of newborns might come from their biological mothers [25]. From the perspective of gut microbiota composition, the dominant phylum of the hen-reared chickens in the first week included *Firmicutes*, *Bacteroidetes*, *Proteobacteria*, *Actinomycetes*, and *Fusobacteria*, which were also abundant in the gut microbiota of the hens. The microbiota of the separately reared chicks was dominated by representatives of phylum *Firmicutes* at all time points examined in the present study. The composition of the gut microbiota of the hen-raised chicks and their mothers were more similar in comparison to the separately reared chicks, which suggests that hen feeding has a profound influence on the establishment of the initial gut microbiota in chicks.

A recent study on the gut microbiota of passerines demonstrated that the oral cavity microbiota of birds has a strong promoting effect on the establishment of their nestlings’ early gut microbiota as a probable consequence of the nestlings being fed by the birds through oral means [19]. Another study indicated that the similar gut microbial communities found in mothers and their nestlings may be due to vertical transmission through feeding [26]. Unlike passerines, hens do not feed their chicks through oral means; instead, the chicks peck food on the ground which is likely to be contaminated with their hen’s feces. We found that the maternal oral microbiota contributed little to the chick’s intestinal community at any stage. However, the hen gut microbiota, followed by feathers, was the most important source of a chick’s gut microbiota in early life (Fig. 4). The fact that chicks peck food contaminated with hen droppings on the ground increases the possibility of transmission of the maternal gut microbiota to the chicks. Chicks often hide under hen feathers to keep warm and thus have full contact with their hen’s feathers, which potentially results in the transfer of bacteria from the feathers to the chick gut.

Ecological theory suggests that microorganisms that pre-empt an ecological niche can affect the health of the host and the phenomenon is known as the “priority effect” [25]. In mammals, there is an overwhelming amount of evidence, associated with the development of the mammalian immune system in early life, which suggests that maternal microbes help build the gut microbiota and improve the immunity in infants [27, 28]. Birds separated from their parents, such as juvenile ostriches, fail to establish a balanced intestinal microflora that leads to increased mortality [29]. Our study revealed that the microbiota of hen-reared chicks had a greater diversity and higher proportion of potential health-associated bacteria, such as the genus *Bacteroides* [30], family *Muribaculaceae* (S24-7) [31, 32] and *Fusobacteriaceae* [33, 34], genus *Turicibacter* [29], and order *Bacillales* [35]. We speculate that this promises to have a beneficial impact on the development of the immune system in the chicks. PICRUSt predicted a greater risk of infectious diseases in the separately reared group of chicks (Figure S1). The H9N2 infection experiment, executed in the present study, supported this. Although the H9N2 infection disturbed the composition of the gut microbiota in both the hen-reared and separately reared groups, the hen-reared group had a relatively more stable gut microbiota post H9N2 infection (Fig. 5C, D, and E). The separately reared group showed drastic changes in the gut microbiota post H9N2 infection, with phyla *Proteobacteria*, *Actinobacteria*, *Bacteroidetes*, and *Fusobacteria* increasing significantly, while the phylum *Firmicutes* decreased sharply at 3 dpi. Our observations are consistent with previous findings that the infection of SPF chickens with H9N2 results in an increase in bacterial members representing the phylum *Proteobacteria* and a decrease in *Firmicutes* [36]. Members of phyla *Bacteroidetes* and *Proteobacteria* have earlier been reported to be increased in mice infected with H9N2 [37, 38]. Changes in gut microbiota can lead to disruption of mucosal immune responses in susceptible hosts [39]. Conversely, more stable gut microbiota might be an important contributory factor in conferring protection and resistance to the host against viral infections. The significant reduction in the phylum *Firmicutes*, which produce short-chain fatty acids, in the separately reared group may affect the bactericidal activity of alveolar macrophages and thus reduce the ability to combat secondary bacterial infections [40]. In our study, we found that the separately reared chicks had a more difficult access to rehabilitation after H9N2 infection. Viral titer positive duration was longer in the oropharyngeal swabs of chicks in the separately reared group. At 7 dpi, contrary to the chicks of the separately reared group, no viruses were detected in the oropharyngeal swabs of chicks in the hen-reared group. Viral titers in the oropharyngeal and cloacal swabs of the separately reared group were higher than in the hen-reared group at 5 dpi, though this difference was not significant.

## Conclusions

In summary, our study investigated the influence of the disruption of microbiota transfer from hens to chicks under current commercial chicken breeding industry practices, where eggs are incubated in a hatchery in the absence of hens. Our analysis demonstrated that hen rearing helps chicks establish a much higher diversity of gut microbiota in early life. On the contrary, the guts of separately reared chicks were colonized by a large number of opportunistic or pathogenic bacteria (e.g., genera *Enterococcus* and *Escherichia*) in early life. Hen-reared chicks retain a relatively stable gut microbiota after H9N2 challenge. These findings advance our understanding of the role of vertical transmission in the initial establishment of neonatal intestinal microbiota of chicks and may be effective in improving the rearing of chicks in the commercial poultry industry.

## Methods

### Experimental animals and sample collection

In the present study, chicks were hatched from SPF White Leghorn chicken eggs. We chose the Qingyuan chicken as the hens because the White Leghorn chicken is not broody. We used three hen-reared (HR) groups and one separately reared (SR) group of chicks. For each hen-reared group, 10 fertile eggs were hatched by a brooding hen, with the newly hatched chicks raised in the presence of the hen. For the separately reared group, 10 fertile eggs were hatched in a cabinet egg incubator at 37.8 °C and 40–50% relative humidity, and the newly hatched chicks were separately reared. Chicks had free access to standard feed and water under a 12-h light and 12-h dark cycle. Cloacal swabs were collected from each chick at days 3, 5, 7, 11, and 17 post-hatching (dph) in all groups. Cloacal swabs, oropharyngeal swabs, and wing feathers were collected from each hen at 3, 5, and 7 dph. All samples were collected and kept on ice before being transported to the laboratory. The samples were immediately frozen at − 80 °C until DNA extraction.

### Avian influenza virus infection experiments

H9N2 avian influenza virus (strain name: A/Chicken/Guangdong/Lz-wzp-10/2013, GenBank accession numbers OK035258 to OK035265) was propagated in 9-day-old SPF chicken embryos, titrated using the hemagglutination assay (HA), and stored at – 80 °C for later use. Chicks were infected with the H9N2 virus at 28 dph. Chicks from a randomly selected hen-reared group and the separately reared group were transferred to an isolator and were inoculated with 10^−6^ 50% embryo infectious dose (EID_50_)/0.2 ml of the H9N2 virus via the ocular and nasal routes. At 3, 5, 7, and 11 days post-infection (dpi), oropharyngeal and cloacal swabs of the chicks were collected and stored in Dulbecco’s Modified Eagle’s Medium (DMEM) containing penicillin–streptomycin at a concentration of 10000u/mL to determine the viral titer. Viral titers of single samples were determined by the EID50 assay and the titer detection limit was 0.75 log10EID50/mL. The Mann-Whitney *U* test was used to compare the differences in viral titers between the hen-reared and separately reared groups. Cloacal swabs of the chicks were collected at 0 (28 dph**)**, 3, 5, 7, and 14 dpi for 16S rRNA sequencing (Table S1**)**.

### DNA extraction and sequencing

Total DNA was extracted using the CTAB/SDS method. PCR amplicons for the V3 and V4 regions of the 16S rRNA gene were generated with the primers 341F (5′-CCTACGGGAGGCAGCAG-3′) and 806R (5′-GGACTACHVGGGTWTCTAAT-3′) [41]. TruSeq® DNA PCR-Free Sample Preparation Kit (Illumina, USA) was used to construct sequencing libraries. Constructed libraries were quantified by Qubit and Q-PCR and sequenced on the Illumina Novaseq 6000 platform. Sequence analysis of the 169 samples from the chick cloacal (*n* = 142), hen cloacal (*n* = 9), hen feather (*n* = 9) and hen oropharyngeal (*n* = 9) swab niches yielded 10,614,968 tags with an average of 62810.46 ± 5307.84 tags per sample.

### 16S rRNA data processing and statistical analysis

Paired-end reads were assigned to samples based on their unique barcode and were truncated by removing the barcode and primer sequences using the Cutadapt (version 1.18) pipeline [42]. Sequences containing ambiguous or low-quality bases were filtered using Trimmomatic [43]. Reads were merged using the FLASH software [44]. Clustering of operational taxonomic units (OTUs) was carried considering a sequence similarity of 97%. Chimeric sequences were removed using Vsearch [45]. We retained OTUs with abundances > 0.01% of the total abundance. Finally, we identified 418 OTUs for the downstream analysis. OTUs were annotated using GreenGenes (v.13.8, 97% identity reference set) [46] and classified at the domain, phylum, class, order, family, genus, and species levels.

Alpha diversity index (Shannon, Observed OTUs) was calculated for the normalized OTU table using the R package vegan (v.2.4.0) and phyloseq (v.1.14.0) [47]. For multiple-group comparisons, the Dunnett test was employed to calculate the significance of the alpha-diversity, with P values corrected using false discovery rate (FDR) correction. The CSS method was used to standardize the OTUs table before calculating the beta diversity. Principal coordinates analysis (PCoA) of the bacterial communities was performed using Bray–Curtis distances. Analysis of similarities (ANOSIM) was performed using the ANOSIM function implemented in the R package vegan, where *R* > 0 suggests that the distance within a group is less than the distance between groups and the groupings are effective.

SourceTracker was used to analyze the possible sources and proportions of the gut microbiota in the early life of the chicks [48]. Linear discriminant analysis (LDA) effect size (LEfSe) was performed using the online Huttenhower Galaxy server (http://huttenhower.sph.harvard.edu/galaxy/). Microbial functional profiles were predicted using PICRUSt [22]. The heatmaps were generated using the TBtools software [49], with the input data being standardized by the R functions scale (*x*, center = TRUE, scale = TRUE).

## Supplementary Information

**Additional file 1: Figure S1**. Microbial metabolic pathways among the hen-reared (HR) and separately-reared (SR) groups. Input data was standardized with the R functions scale (x, center = TRUE, scale = TRUE).

**Additional file 2: Table S1.** Metadata for each sample used in this study.

**Additional file 3: Table S2.** Comparison of the alpha diversity (Observed OTU and Shannon index) between the pairwise groups performed using the Dunnett test.

**Additional file 4: Table S3.** Comparison of the relative abundances (mean ± SEM) of the major bacterial families representing the gut microbiota of the hen-reared (HR 2) group and separately-reared group at various stages from 3 dph to 28 dph.

**Additional file 5: Table S4.** Comparison of the relative abundances (mean ± SEM) of the major bacterial phyla representing the gut microbiota between the pairwise groups of post H9N2 infected chickens by Mann-Whitney U test.

## Data Availability

The datasets generated in the current study were deposited to the NCBISRA database under the BioProject accession no. PRJNA747156.

## Abbreviations

HR: Hen-reared
SR: Separately reared
dph: Days post-hatching
dpi: Days post-infection
HC: Hen cloacal
HF: Hen feathers
HO: Hen oropharyngeal
PCoA: Principal coordinate analysis
LEfSe: Linear discriminant analysis effect size
AIV: Avian influenza virus
HA: Hemagglutination assay
EID50: 50% embryo infectious dose
OTUs: Operational taxonomic units
ANOSIM: Analysis of similarities
