# Supplementary figures and images for: Hen raising helps chicks establish gut microbiota in their early life and improve microbiota stability after H9N2 challenge
Source: Microbiome. 2022 Jan 24;10:14. doi: 10.1186/s40168-021-01200-z (PMC8785444; doi:10.1186/s40168-021-01200-z)

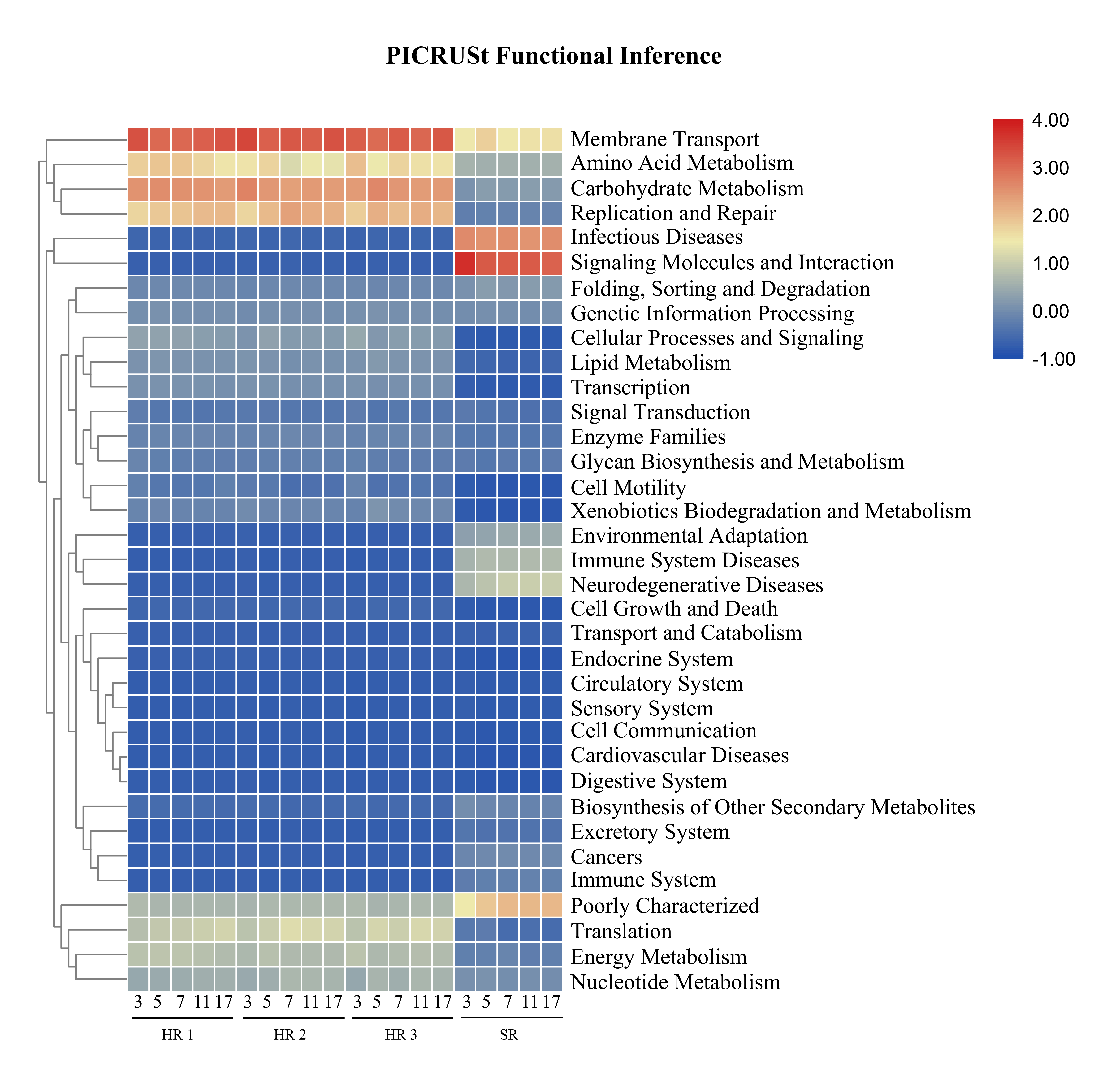

Supplement: Supplementary file 2 — Additional file 1: Figure S1. Microbial metabolic pathways among the hen-reared (HR) and separately-reared (SR) groups. Input data was standardized with the R functions scale (x, center = TRUE, scale = TRUE). [file 40168_2021_1200_MOESM1_ESM.tif]
