# Supplementary material for: Hen raising helps chicks establish gut microbiota in their early life and improve microbiota stability after H9N2 challenge
Source: Microbiome. 2022 Jan 24;10:14. doi: 10.1186/s40168-021-01200-z (PMC8785444; doi:10.1186/s40168-021-01200-z)
Supplement: Supplementary file 3 — Additional file 2: Table S1. Metadata for each sample used in this study. [file 40168_2021_1200_MOESM2_ESM.docx]

**Table S1** Metadata for each sample used in this study.

| Stage | _day_ ^group^ | SR | HR 1 | | | | HR 2 | | | | HR 3 | | | |
| --- | --- | --- | --- | --- | --- | --- | --- | --- | --- | --- | --- | --- | --- | --- |
|  |  | chicks | chicks | hen | | | chicks | hen | | | chicks | hen | | |
| Feeding |  | cloacal swab | cloacal swab | cloacal swab | feather | OP  swab | cloacal swab | cloacal swab | feather | OP swab | cloacal swab | cloacal swab | feather | OP  swab |
|  | 3 dph | 5 | 5 | 1 | 1 | 1 | 5 | 1 | 1 | 1 | 5 | 1 | 1 | 1 |
|  | 5 dph | 5 | 5 | 1 | 1 | 1 | 5 | 1 | 1 | 1 | 5 | 1 | 1 | 1 |
|  | 7 dph | 4 | 5 | 1 | 1 | 1 | 5 | 1 | 1 | 1 | 5 | 1 | 1 | 1 |
|  | 11 dph | 5 | 5 |  |  |  | 5 |  |  |  | 5 |  |  |  |
|  | 17 dph | 5 | 5 |  |  |  | 4 |  |  |  | 5 |  |  |  |
|  |  | HR chicks  (HR 2) | SR chicks |  |  |  |  |  |  |  |  |  |  |  |
| Infection | 28 dph (0 dpi ) | 4 | 5 |  |  |  |  |  |  |  |  |  |  |  |
|  | 3 dpi | 5 | 5 |  |  |  |  |  |  |  |  |  |  |  |
|  | 5 dpi | 5 | 5 |  |  |  |  |  |  |  |  |  |  |  |
|  | 7 dpi | 4 | 4 |  |  |  |  |  |  |  |  |  |  |  |
|  | 14 dpi | 3 | 4 |  |  |  |  |  |  |  |  |  |  |  |

Note: HR, hen-reared group; SR, separately-reared group; OP swab, oropharyngeal swab.
