# Supplementary material for: Hen raising helps chicks establish gut microbiota in their early life and improve microbiota stability after H9N2 challenge
Source: Microbiome. 2022 Jan 24;10:14. doi: 10.1186/s40168-021-01200-z (PMC8785444; doi:10.1186/s40168-021-01200-z)
Supplement: Supplementary file 4 — Additional file 3: Table S2. Comparison of the alpha diversity (Observed OTU and Shannon index) between the pairwise groups performed using the Dunnett test. [file 40168_2021_1200_MOESM3_ESM.docx]

**Table S2** Comparison of the alpha diversity (Observed OTU and Shannon index) between the pairwise groups by Dunnett test.

| day | group | Observed | | | | | Shannon | | | | |
| --- | --- | --- | --- | --- | --- | --- | --- | --- | --- | --- | --- |
|  |  | Estimate | Std.Error | t | value | Pr(>\|t\|) | Estimate | Std.Error | t | value | Pr(>\|t\|) |
| dph3 | HR1-SR==0 | 89.2 | 31.47 | 2.835 | 0.0309 | * | 1.5498 | 0.5243 | 2.956 | 0.0245 | * |
|  | HR2-SR==0 | 28 | 31.47 | 0.89 | 0.7075 |  | 0.3405 | 0.5243 | 0.649 | 0.8536 |  |
|  | HR3-SR==0 | 100.8 | 31.47 | 3.203 | 0.0147 | * | 2.3759 | 0.5243 | 4.531 | <0.001 | *** |
| dph5 | HR1-SR==0 | 133.2 | 20.14 | 6.613 | <0.001 | *** | 1.3817 | 0.5266 | 2.624 | 0.0471 | * |
|  | HR2-SR==0 | 54 | 20.14 | 2.681 | 0.042 | * | 0.7359 | 0.5266 | 1.397 | 0.3892 |  |
|  | HR3-SR==0 | -3.8 | 20.14 | -0.189 | 0.995 |  | -0.6526 | 0.5266 | -1.239 | 0.4806 |  |
| dph7 | HR1-SR==0 | 127.3 | 31.98 | 3.981 | 0.00352 | ** | 1.6862 | 0.5679 | 2.969 | 0.0244 | * |
|  | HR2-SR==0 | -7.1 | 31.98 | -0.222 | 0.9914 |  | -0.4641 | 0.5679 | -0.817 | 0.7425 |  |
|  | HR3-SR==0 | 69.7 | 31.98 | 2.18 | 0.10768 |  | 0.6655 | 0.5679 | 1.172 | 0.5104 |  |
| dph11 | HR1-SR==0 | 92.2 | 27.04 | 3.409 | 0.00969 | ** | 0.5159 | 0.7326 | 0.704 | 0.823 |  |
|  | HR2-SR==0 | 48.2 | 27.04 | 1.782 | 0.21675 |  | -0.444 | 0.7326 | -0.606 | 0.876 |  |
|  | HR3-SR==0 | 33.2 | 27.04 | 1.228 | 0.48775 |  | -0.5319 | 0.7326 | -0.726 | 0.81 |  |
| dph17 | HR1-SR==0 | 18.8 | 36.1 | 0.521 | 0.917 |  | -0.02194 | 0.57146 | -0.038 | 1 |  |
|  | HR2-SR==0 | -24.2 | 38.29 | -0.632 | 0.865 |  | -0.52083 | 0.60613 | -0.859 | 0.732 |  |
|  | HR3-SR==0 | 36.8 | 36.1 | 1.019 | 0.627 |  | 0.1293 | 0.57146 | 0.226 | 0.992 |  |
| dph28 | HR2-SR==0 | 9.20 | 13.85 | 0.664 | 0.528 |  | 0.8141 | 0.3530 | 2.306 | 0.0545 |  |

Note: HR, hen-reared group; SR, separately-reared group.
