# Supplementary material for: Hen raising helps chicks establish gut microbiota in their early life and improve microbiota stability after H9N2 challenge
Source: Microbiome. 2022 Jan 24;10:14. doi: 10.1186/s40168-021-01200-z (PMC8785444; doi:10.1186/s40168-021-01200-z)
Supplement: Supplementary file 5 — Additional file 4: Table S3. Comparison of the relative abundances (mean ± SEM) of the major bacterial families representing the gut microbiota of the hen-reared (HR 2) group and separately-reared group at various stages from 3 dph to 28 dph. [file 40168_2021_1200_MOESM4_ESM.docx]

**Table S3.** Comparison of the relative abundances (mean ± SEM) of the major bacterial families representing the gut microbiota of the hen-reared (HR 2) group and separately-reared group at various stages from 3 dph to 28dph.

| Bacterial family | HR VS SR  (3dph) | | HR VS SR  (5dph) | | HR VS SR  (7dph) | | HR VS SR  (11dph) | | HR VS SR  (17dph) | | HR VS SR  (28dph) | |
| --- | --- | --- | --- | --- | --- | --- | --- | --- | --- | --- | --- | --- |
|  | mean ± SEM | P-values | mean ± SEM | P-values | mean ± SEM | P-values | mean ± SEM | P-values | mean ± SEM | P-values | mean ± SEM | P-values |
| *Lactobacillaceae* | 0.402±0.353  VS 0.080±0.0187 | 0.144 | 0.435±0.269 VS 0.378±0.184 | 0.835 | 0.815±0.108  VS  0.638±0.233 | 0.391 | 0.699 ± 0.245  VS  0.539 ± 0.300 | 0.296 | 0.695±0.157 VS 0.567±0.339 | 0.713 | 0.090±0.087  VS  0.046±0.031 | 0.903 |
| *Enterococcaceae* | 0.390±0.293 VS 0.637±0.268 | 0.144 | 0.019±0.009 VS 0.174±0.065 | **0.012** | 0.022±0.008  VS  0.105±0.064 | **0.020** | 0.046 ± 0.029  VS 0.051±0.028 | 0.676 | 0.025±0.020 VS 0.092±0.057 | 0.06 | 0.593±0.033  VS  0.194±0.295 | 0.178 |
| *Lachnospiraceae* | 0.024±0.009  VS 0.023±0.021 | 1.000 | 0.052±0.066 VS 0.090±0.097 | 0.403 | 0.013±0.0150 VS  0.065±0.055 | **0.037** | 0.020 ± 0.020  VS  0.127 ± 0.116 | **0.037** | 0.065±0.074 VS 0.092±0.087 | 0.391 | 0.012±0.010  VS  0.006±0.002 | 0.391 |
| *Ruminococcaceae* | 0.043±0.062 VS 0.014±0.017 | 0.210 | 0.074±0.080 VS 0.027±0.021 | 0.676 | 0.006±0.003  VS 0.024±0.018 | 0.111 | 0.013 ± 0.016  VS  0.057 ± 0.060 | 0.060 | 0.011±0.017 VS 0.039±0.036 | 0.111 | 0.013±0.009  VS  0.007±0.003 | 0.270 |
| *Clostridiaceae* | 0.011±0.012 VS 0.018±0.018 | 0.296 | 0.033±0.035 VS 0.181±0.250 | 0.296 | 0.011±0.015  VS  0.016±0.014 | 0.391 | 0.006 ± 0.006  VS 0.013±0.013 | 0.531 | 0.008±0.010 VS 0.005±0.003 | 0.713 | 0.086±0.076  VS  0.201±0.378 | 1.00 |
| *Bacteroidaceae* | 0.009±0.0137 VS 0.003±0.004 | 0.403 | 0.079±0.129 VS 0.003±0.003 | 0.060 | 0.006±0.010  VS  0.007±0.008 | 0.713 | 0.014 ± 0.022  VS  0.025±0.028 | 0.531 | 0.013±0.015 VS 0.055±0.101 | 0.540 | 6.4E-04±7.2E-04 VS 6.7E-05±3.9E-05 | 0.270 |
| *Erysipelotrichaceae* | 0.022±0.023 VS 0.065±0.096 | 0.676 | 0.018±0.018 VS 0.029±0.023 | 0.403 | 0.003±0.003  VS  0.016±0.013 | **0.020** | 0.003 ± 0.004  VS 0  .017 ± 0.014 | 0.060 | 0.002±0.002 VS 0.004±0.002 | 0.270 | 0.002±0.004  VS 3.2E-04±2.4E04 | 0.270 |
| *Fusobacteriaceae* | 0.031±0.064 VS 0.001±0.001 | **0.037** | 0.073±0.113 VS 0.001±0.001 | **0.037** | 4.9E-04±2.8E-04 VS 4.3E-04±2.9E-04 | 0.713 | 0.008 ± 0.010  VS  0.001 ± 0.002 | 0.095 | 0.001±0.001 VS 0.001±0.001 | 0.391 | 9.7E-06±1.1E-05 VS 1.5E-05±1.5E-05 | 0.701 |

Note: P-values were calculated with a Mann-Whitney U test. Significant differences are marked in bold.

HR, hen-reared group; SR, separately-reared group.
