# Supplementary material for: Hen raising helps chicks establish gut microbiota in their early life and improve microbiota stability after H9N2 challenge
Source: Microbiome. 2022 Jan 24;10:14. doi: 10.1186/s40168-021-01200-z (PMC8785444; doi:10.1186/s40168-021-01200-z)
Supplement: Supplementary file 6 — Additional file 5: Table S4. Comparison of the relative abundances (mean ± SEM) of the major bacterial phyla representing the gut microbiota between the pairwise groups of post H9N2 infected chickens by Mann-Whitney U test. [file 40168_2021_1200_MOESM5_ESM.docx]

**Table S4.** Comparison of the relative abundances (mean ± SEM) of the major bacterial phyla representing the gut microbiota between the pairwise groups of post H9N2 infected chickens by Mann-Whitney U test.

| Group | Bacterial phyla | 28 dph VS 3dpi | | 3 dpi VS 5dpi | | 5 dpi VS7dpi | | 7 dpi VS 14dpi | |
| --- | --- | --- | --- | --- | --- | --- | --- | --- | --- |
|  |  | mean ± SEM | P-values | mean ± SEM | P-values | mean ± SEM | P-values | mean ± SEM | P-values |
| HR group | *Actinobacteria* | 0.003 ± 0.003  VS  0.009 ± 0.004 | 0.067 | 0.009±0.004  VS  0.021±0.013 | 0.210 | 0.021±0.013  VS  0.026±0.018 | 0.902 | 0.026±0.018  VS  0.007±0.003 | 0.216 |
|  | *Bacteroidetes* | 0.009 ± 0.016  VS  0.017 ± 0.009 | 0.270 | 0.017±0.009  VS  0.075±0.121 | 0.676 | 0.075±0.121  VS  0.007±0.007 | 0.540 | 0.007±0.007  VS  0.011±0.018 | 0.860 |
|  | *Cyanobacteria* | 4.0E-04 ±6.2E-04  VS  0.003 ± 0.005 | 0.270 | 0.003±0.005  VS  0.008±0.016 | 0.531 | 0.008±0.016  VS  0.002±0.002 | 0.713 | 0.002±0.002  VS  1.6E-04±2.0E-04 | 0.285 |
|  | *Firmicutes* | 0.891 ± 0.187  VS  0.942 ± 0.031 | 0.391 | 0.942±0.031  VS  0.804±0.197 | 0.403 | 0.804±0.197  VS  0.949±0.019 | 0.540 | 0.949±0.019  VS  0.936±0.027 | 0.860 |
|  | *Fusobacteria* | 9.7E-06 ± 1.1E-05  VS  0.003 ± 0.004 | 0.105 | 0.003±0.004  VS  0.024±0.051 | 0.531 | 0.024±0.051  VS  6.2E-04±7.7E-04 | 0.391 | 6.2E-04±7.7E-04 VS  2.3E-04±2.9E-04 | 0.596 |
|  | *Proteobacteria* | 0.095 ± 0.174  VS  0.026 ± 0.022 | 0.713 | 0.026±0.022  VS  0.069±0.115 | 1.00 | 0.069±0.115  VS  0.015±0.005 | 0.391 | 0.015±0.005  VS  0.041±0.004 | 0.052 |
|  | *Synergistetes* | 3.2E-05 ± 4.0E-05  VS  1.6E-04 ± 3.0E-04 | 0.709 | 1.6E-04±3.0E-04  VS  1.0E-04±1.4E-04 | 0.590 | 1.0E-04±1.4E-04 VS  9.7E-06±1.1E-05 | 0.893 | 9.7E-06±1.1E-05 VS 6.7E-05±1.0E-04 | 0.854 |
|  | TM7 | 0.001 ± 0.002  VS  4.1E-05 ± 9.1E-05 | 0.227 | 4.1E-05±9.1E-05  VS  1.1E-05±2.4E-05 | 1.00 | 1.1E-05±2.4E-05 VS  0±0 | 0.502 | 0±0  VS  0±0 | NA |
|  | *Verrucomicrobia* | 4.1E-05 ± 8.3E-05  VS  3.4E-06 ± 7.6E-06 | 0.867 | 3.4E-06±7.6E-06  VS  3.8E-05±5.6E-05 | 0.441 | 3.8E-05±5.6E-05 VS  0±0 | 0.240 | 0±0  VS  0.005±0.008 | 0.123 |
|  |  |  |  |  |  |  |  |  |  |
|  | Bacterial phyla | 28 dph VS 3 dpi | | 3 dpi VS 5dpi | | 5 dpi VS7dpi | | 7 dpi VS 14dpi | |
|  |  | mean ± SEM | P-values | mean ± SEM | P-values | mean ± SEM | P-values | mean ± SEM | P-values |
| SR group | *Actinobacteria* | 0.014 ± 0.021  VS  0.078  ± 0.062 | **0.037** | 0.078  ± 0.062  VS  0.113 ± 0.068 | 0.531 | 0.113± 0.068  VS  0.015 ± 0.010 | **0.037** | 0.015± 0.010  VS  0.007 ± 0.004 | 0.112 |
|  | *Bacteroidetes* | 2.7E-04± 3.4E-04  VS  0.127  ± 0.108 | **0.012** | 0.127  ± 0.108  VS  0.038 ± 0.056 | 0.144 | 0.038 ± 0.056  VS  0.008 ± 0.009 | 0.391 | 0.008 ± 0.009  VS  0.002 ± 0.001 | 0.470 |
|  | *Cyanobacteria* | 2.6E-04  ± 3.3E-04  VS  4.0E-04  ± 2.3E-04 | 0.403 | 4.0E-04  ± 2.3E-04  VS  0.003± 0.005 | 0.403 | 0.003 ± 0.005  VS  4.6E-04 ± 4.4E-04 | 0.391 | 4.6E-04 ± 4.4E-04  VS  0.001 ± 0.001 | 0.312 |
|  | *Firmicutes* | 0.983  ± 0.022  VS  0.529  ± 0.180 | **0.012** | 0.529  ± 0.180  VS  0.802 ± 0.105 | 0.095 | 0.802 ± 0.105  VS  0.968± 0.015 | **0.020** | 0.968 ± 0.015  VS  0.984 ± 0.006 | 0.312 |
|  | *Fusobacteria* | 1.5E-05  ± 1.5E-05  VS  0.140  ± 0.209 | **0.012** | 0.140 ± 0.209  VS  0.005± 0.004 | **0.037** | 0.005 ± 0.004  VS  0.001 ± 0.002 | 0.270 | 0.001 ± 0.002  VS  2.2E-04 ± 2.4E-04 | 0.312 |
|  | *Proteobacteria* | 0.002 ± 0.001  VS  0.118  ± 0.074 | **0.012** | 0.118  ± 0.074  VS  0.038 ± 0.025 | **0.037** | 0.038 ± 0.025  VS  0.008 ± 0.005 | **0.020** | 0.008 ± 0.005  VS  0.007 ± 0.004 | 0.665 |
|  | *Synergistetes* | 0  ± 0  VS  0.003  ± 0.005 | **0.007** | 0.003  ± 0.005  VS  2.6E-04± 2.6E-04 | 0.094 | 2.6E-04 ± 2.6E-04  VS  3.4E-04 ± 4.8E-04 | 0.901 | 3.4E-04 ± 4.8E-04  VS  1.0E-05 ± 1.2E-05 | 0.645 |
|  | TM7 | 2.5E-05  ± 3.6E-05  VS  0.005  ± 0.010 | **0.020** | 0.005 ± 0.010  VS  8.2E-05 ± 7.7E-05 | 0.094 | 8.2E-05 ± 7.7E-05  VS  1.1E-04 ± 2.0E-04 | 0.898 | 1.1E-04 ± 2.0E-04  VS  0 ± 0 | 0.186 |
|  | *Verrucomicrobia* | 0  ± 0  VS  1.4E-04 ± 2.6E-04 | 0.072 | 1.4E-04 ± 2.6E-04  VS  1.5E-05 ± 2.3E-05 | 0.504 | 1.5E-04± 2.3E-04  VS  2.1E-05 ± 3.1E-05 | 0.893 | 2.1E-05 ± 3.1E-05  VS  0 ± 0 | 0.186 |

Note: NA, indicate that the relative abundances of phyla were undetectable. Significant differences are marked in bold.

HR, hen-reared group; SR, separately-reared group.
